# Development of methods for WHO quality standards for child and youth mental health services to improve quality of care and patient safety in the WHO European region

**DOI:** 10.3389/frhs.2025.1644419

**Published:** 2025-10-02

**Authors:** Jennifer Hall, Katie H. Atmore, Rimma Belikova, Chris Boyd-Skinner, Lisa Corrigan, Anastasia Giannaki, Ana Isabel F. Guerreiro, Penny Kalpaxi, Colette Kelly, Konstantinos Kotsis, Dennis Ougrin, Catalina Popoviciu, Anne Marie Råberg Christensen, Dion Ras, Cassie Redlich, Tatiana Taylor Salisbury, Raffaella Sibilio, Ana Maria Tijerino, Ledia Lazeri, Joao Breda

**Affiliations:** ^1^ World Health Organization Office on Quality of Care and Patient Safety (Greece), World Health Organization (Greece), Athens, Greece; ^2^ University College London Institute for Global Health, London, United Kingdom; ^3^ Methodological Centre, Children’s Clinical University Hospital Latvia, Riga, Latvia; ^4^ Department of Health, Ireland, Dublin, Ireland; ^5^ Independent Expert, Strasbourg, France; ^6^ University of Galway Health Promotion Research Centre, Galway, Ireland; ^7^ Community CAMHS, Department of Psychiatry, Faculty of Medicine, School of Health Sciences, University of Ioannina, Ioannina, Greece; ^8^ Youth Resilience Unit, WHO Collaborating Centre for Mental Health Services Development, Centre for Psychiatry and Mental Health, Queen Mary University of London Wolfson Institute of Population Health, London, United Kingdom; ^9^ East London NHS Foundation Trust, London, United Kingdom; ^10^ Let’s CEE- A Youth Mental Health Initiative for the Central and Eastern European Region, Bucharest, Romania; ^11^ Independent, Copenhagen, Denmark; ^12^ International Association for Youth Mental Health, Meerssen, Limburg, Netherlands; ^13^ Mental Health Flagship, World Health Organization Regional Office for Europe, Copenhagen, Denmark; ^14^ Health Service and Population Research Department, King’s College London Institute of Psychiatry Psychology & Neuroscience, London, United Kingdom

**Keywords:** child and adolescent mental health (CAMH), child and adolescent mental health care, quality of care (measurement), quality standards and criteria, quality of care (QoC)

## Abstract

**Background:**

Child and youth mental health care is of varying quality across the WHO European Region, with many settings being low-resourced. To improve and standardize quality of care, WHO Regional Office for Europe is developing quality standards for child and youth mental health services. This research aims to develop evidence informed methods to develop these quality standards.

**Methods:**

Desk reviews of grey literature aimed to understand what approaches have been used or recommended to develop quality standards for child and youth mental health/health for use across a range of different countries, and consultation was sought from an expert steering group. A thematic approach was used to synthesize relevant themes. The methods were developed based on the results of these steps.

**Results:**

Desk reviews identified variation in approaches used and recommended to develop quality standards, with limited available guidance applicable across different resource settings. Nine key themes from stakeholder consultations were highlighted. Based on these results, a seven-step methodology was created to develop the quality standards for child and youth mental health which prioritizes using an evidence-based approach and inputs from a wide range of stakeholders.

**Discussion:**

The methods taken to develop quality standards need to be rigorous to ensure that standards accurately define high-quality care for a service. There is a need to develop a unified approach to developing quality standards. It is hoped that this paper will provide inspiration for others developing quality standards for child and youth mental health services and spark research in this area.

## 1 Introduction

Child and youth mental health is a growing concern in the World Health Organization (WHO) European Region (1) (“the Region”). One in five adolescents aged 15–19 years old (2) are living with a mental health condition, and suicide is the leading cause of death for young people aged 15–29 years old (3). Mental health treatments and interventions provide the opportunity to prevent the onset and escalation of mental health conditions across the life course (4), having positive implications for individuals, families and communities and reducing costs for society as a whole (5).

Many children and young people requiring mental health support do not access the care they need. For those who do access care, inequities in the quality of care received exist. Quality health services should be effective, safe, people-centered, timely, equitable, integrated and efficient (6). Hence patient safety is a core component of quality of care. Challenges to patient safety in child and youth mental health care can arise from ineffective engagement, ineffective practice and adverse events (7). Sources of potential harm in mental health treatment for children and young people include unsafe psychological therapies (8) and unsafe psychotropic treatment (9). For those in inpatient care, potential harm can results from the use of physical restraint (10) and longer admissions which have been associated with increased levels of self-harm (11). High-quality care is also impacted by other factors, such as the number and types of services available, of which there is considerable variation across the Region (12). Hence, there is need to standardize mental health care quality across the Region to improve outcomes for children and young people.

Although there have been calls for parity between physical and mental health care (13), this has not become a reality across the Region: the median percentage of the health budget spent on mental health overall was 3.60% (12), and the median number of health workers in mental health was 4.48 per 10,000 population (12), a small fraction of the median of 130.3 health workers overall per 10,000 population (14). The situation is more critical for child and adolescent mental health, where there are just 1.25 health workers per 10,000 population (12). Despite all countries in the Region being classified as either high or middle income (15), most, if not all, have reported having inadequate resources for child and youth mental health care with multiple calls for more investment in this area (16). Thus, we argue that many—if not all- countries are under-resourced when it comes to child and youth mental health.

One way to reduce inequities and inconsistencies in care is through setting evidence-based quality standards, guidelines and protocols, to lay the framework for quality assessment and improvement (17). At its first meeting, the WHO Pan-European Mental Health Coalition identified reducing inequities in child and youth mental health care through the development of quality standards as a priority (18). Only a few high-income countries in the Region have developed quality standards for child and youth mental health care [e.g., the United Kingdom (19)] and quality initiatives remain concentrated in this handful of high-income countries (20). Hence developing quality standards for child and youth mental health services is the first step to define high-quality care for this group and standardize care quality across the Region.

The relevance and representation of the quality standards depends on the methods used for their development. We aimed to develop quality standards that can be applied across multiple settings, are evidence-based and are applicable in all countries without exception.

We previously conducted a rapid systematic review of published scientific articles to understand whether a unified approach to develop quality standards and indicators for mental health had been detailed in scientific literature. The results will be published elsewhere and showed that no one “gold standard” approach was used that could be easily adapted for our purposes and is consistent with other research (21).

This research aimed to develop robust methods grounded in available evidence for developing quality standards for child and youth mental health across the Region. In the development of our methods, we sought to answer the following question:

*Based on existing guidance and experience, what methods should be used to develop quality standards for child and youth mental health services in the WHO European Region?*

Our previously conducted rapid systematic review of scientific articles failed to identify a unified approach to developing quality standards for mental health. These will be published and are described in more detail elsewhere. Therefore, this research focuses on grey literature and stakeholder consultation, addressing three objectives:

(i) To understand what approaches have been used by international organizations to develop quality standards for use across a wide range of different settings in child and youth health or mental health;
(ii) To understand what methods have been recommended to develop quality standards for health services across different resource settings; and
(iii) To develop a method for the development of quality standards for child and youth mental health services across the WHO European Region.

It is hoped that this will provide a proposed model to guide the development of future quality standards across contexts of varying resources.

## 2 Methods

To achieve the research objectives, a mixed-method approach was used. A desk review of grey literature related to the development of quality standards was conducted. The results of the desk searchers were triangulated with insights from expert consultation to develop the final methods for the development of the quality standards for child and youth mental health services.

### 2.1 Grey literature desk review

#### 2.1.1 Search strategy

The websites of international organizations that hold responsibility for health/mental health and children and young people were searched with terms relating to mental health, health, quality standards, guidance, methods, and health and children and adolescents. The following websites and databases were searched:

- WHO global
- WHO Regional websites
- United Nations International Children's Emergency Fund (UNICEF)
- The Organization for Economic Co-operation and Development
- European Commission
- World Economic Forum
- International Society for Quality in Health Care
- Institute for Healthcare Improvement

Results were supplemented by a Google search. Searches were conducted between September 2023 and July 2024 by the primary researcher (JH).

#### 2.1.2 Eligibility criteria

Different eligibility criteria were applied for the two main objectives of the desk searches.

(i) To understand what approaches have been used by international organizations to develop quality standards for use across a wide range of different settings in child and adolescent health or mental health.

Literature relating to quality standards for use across a wide variety of resource settings and for child and adolescent health or mental health services was included as these were considered of most relevance to our research question. See below for inclusion and exclusion criteria.

*Inclusion*

- Outlined methods to develop quality standards.
- The quality standards are meant to be used across a variety of different resource settings.
- The quality standards are aimed at either child and adolescent health settings or mental health settings.
- Published in English.

*Exclusion*

- No methods were mentioned in the document.
- The quality standards are meant to be used in a single country or service.
- The quality standards are aimed at other health services/topics to child and adolescent health settings or mental health settings.
- Published in another language to English.
- To understand what methods have been recommended to develop quality standards for health services across different resource settings.

Guidance documents developed for use by other stakeholders were included, and documents that outlined methods used to develop a specific set of quality standards but were not guidance documents were excluded. Since developing quality standards in healthcare is a different process from developing quality standards in other industries, it was decided to include only guidance relating to healthcare.

See below for full inclusion and exclusion criteria.

*Inclusion*

- Recommended methods to develop quality standards for use by other stakeholders.
- For use in developing quality standards for healthcare.
- Published in English.

*Exclusion*

- Outlined methods used to develop the organization's own quality standards, but not recommended methods for others.
- Outlined recommended methods to develop quality standards in other industries rather than healthcare.
- Published in another language to English.

#### 2.1.3 Data extraction and synthesis

A data extraction framework was adapted from proposed steps to develop quality indicators for global health (21) (see Tables 1, 2). An additional element on user participation was added due its high priority in the WHO Regional Office for Europe work on child and youth mental health (22).

**Table 1:** Data extraction from included grey literature which outlines methods taken to develop quality standards for use across a wide range of different settings in child and youth health or mental health [objective (i)].

Document	Preparatory work	Methods used to identify potential quality standards	Methods used to prioritize and define quality standards	Pilot/implementation	Inputs from users
WHO-UNAIDS Global Standards for Quality Health Care Services for Adolescents (22)	Not mentioned	- Literature review of published and unpublished literature on existing facilitators and barriers to improving the quality of health care for adolescents - Global survey with primary care providers - Global survey with adolescents - Analysis of national standards from 25 countries	1. Review by the technical working group 2. Peer review – WHO, governments, academia, NGOs, development partners	Field test and consolidation phase	Global surveys included adolescents and primary care providers.
European Standards & Indicators for Health Promoting Schools (23)	Not mentioned	Databases, key journals, and documents provided by the Schools for Health in Europe (SHE) National Coordinators were screened, resulting in 95 statements.	1. Statements were categorized into a conventional content analysis, which resulted in 15 areas of the Health Promoting Schools Framework and 10 cores standards. 2. Synthesis was made to result in 8 European HPS Standards. 3. A semi structured survey was used to gather knowledge and expertise from the SHE coordinators and Research Group members about what should be included (*n* = 31 responses). 4. The survey results were used to refine the standards	Not specified	Not mentioned.

**Table 2:** Data extraction from included grey literature which outlines recommended methods to develop quality standards for health services across different resource settings [objective (ii)].

Document	Preparatory work	Methods used to identify potential quality standards	Methods used to prioritize and define quality standards	Pilot/implementation	Inputs from users
WHO Making health services adolescent friendly. Developing national quality standards for adolescent-friendly health services—(25)	1. Understand the perspective of key stakeholders to explain the WHO approach to promoting adolescent health. 2. Establish the basis for formulating the national quality standards – through policies and strategies. 3. Examine the programmatic implications of applying the quality standards—understand the situation, quality improvement principles and what is needed to implement the standards. 4. Obtain public support of stakeholders necessary for implementation of quality standards	1. Formulate the desired quality to be achieved through the standards 2. Identify input, process and output criteria to achieve each standard 3. Identify actions needed at national, district and local level to achieve each criteria. 4. Identify the indicators to measure the achievement of the criteria and means for verifying these. 5. Ensure that the draft standards and accompanying elements are cleared by relevant authorities after being reviewed and revised, as needed.	Not specified	1. Develop an implementation guide that outlines what district health management teams and managers of health facilities need to do to ensure that the structure criteria accompanying each standard statement are in place. 2. Develop a monitoring guide that outlines what and how district health management teams and managers of health facilities need to track implementation.Inform key stakeholders at the nitinol level, how could help or hinder the implementation of the initiative; explain what it aims to achieve and how it aims to do this.	Not specified
WHO Quality Improvement for Mental Health (2003) (29)	1. Mentions the importance to develop committee/ working group with relevant stakeholders	1. Guidance states to decide the format for standards, including what domains they cover and the criteria to measure each standards.	Not specified	Not specified	Mentions that all relevant stakeholders should be included
National Institute for Health and Care Excellence (2014). Principles for developing clinical Quality Standards in low and middle income countries (28)	1. Convene a quality standards development committee 2. Prioritize the topic areas for the quality standard 3. Recruit a Working Group	1. Select Source documents, e.g., international clinical guidelines. Quality standards should be based on evidence-informed recommendations. 2. Select recommendations from source documents 3. Draft the Quality Standard based on the guidance recommendations where there is evidence that current practice does not align with the recommendations. Statements cover areas where quality can be improved. 4. Define structure, process, and outcome measures for the standards.	1. Draft quality standard is updated based on consultation with the Working Group, 2. Wider consultation with interested parties (interviews, surveys or workshops). 3. Conduct a budget impact analysis to consider the costs of implementing the changes. 4. Approve and publish the quality standard.	1. Pre implementation tasks: planning workshop to discuss implementation plan. 2. Pilot the quality standard.	States service users (patients and carers) should be involved in the working group

### 2.2 Consultation with experts

#### 2.2.1 Recruitment

An expert steering group was set up, consisting of people with professional expertise in child and youth mental health, young people with lived experience expertise of child and youth mental health services, and people with experience of developing and/or implementing quality standards for child and youth health or mental health.

To recruit people who met this criteria, a two pronged approach was taken:

1. A call-out to the WHO Regional Office for Europe Pan European Mental Health Coalition.
2. A request for recommendations from WHO staff members.

This resulted in twenty people with expertise either in developing quality standards for child and youth health/mental health, or lived experience of child and youth mental health services were identified to take part in the expert steering group.

#### 2.2.2 Data collection

Data was collected from this group in a number of different ways:

1. Individual interviews (*n* = 16) to learn from their experience of developing quality standards for child and youth health or child and adolescent/youth mental health services, and to understand any challenges, barriers and/or recommendations to be aware of. Detailed written notes were taken during the interview, and written up immediately.
2. A draft version of the methods document was sent out for written review by the whole steering group and updated. Written feedback was collated.
3. A hybrid in person/virtual two day meeting was held in Athens, Greece, where the results from the initial phases of developing the methods were reviewed in more detail and the processes were discussed. Detailed written notes were taken during the meeting, and summarized in a meeting report which was sent around for review to all meeting participants (*n* = 14).
4. An updated version of the methods was then sent around for final review and was revised based on written feedback (*n* = 6).

#### 2.2.3 Data synthesis

Data synthesis aimed to extract themes to inform methods to develop our quality standards for child and youth mental health services. Due to time and resource restraints, a thorough thematic analysis [e.g., as outlined by Braun and Clarke (23)] was not possible. Hence a pragmatic approach was taken. Written notes from meetings and written feedback on the methods documents were reviewed, and key themes were coded and identified by the first author (JH). These themes were summarized and sent around to the expert steering group for review, and updated accordingly.

### 2.3 Writing of methods for WHO quality standards for child and youth mental health services

The data collated from steps (1) and (2), as well as the previously conducted rapid review of scientific literature on methods taken to develop quality standards and indicators for mental health were used to iteratively write and refine the methods document. This was done in parallel with steps (1) and (2).

## 3 Results

### 3.1 Desk reviews

*Objective (i): To understand what approaches have been used by international organizations to develop quality standards for use across a wide range of different settings in child and youth health or mental health.*

Initial searches yielded four documents that outline quality standards for child and youth health or child and youth mental health (24–27). After full text review, two were excluded (24, 25) due to not detailing the methods taken. A description of data extraction from the remaining two documents (26, 27) is provided in Table 1.

Neither document outlined the preparatory work undertaken before developing the quality standards (26, 27). Both documents outlined a process of identifying and prioritizing quality standards (26, 27). To identify potential quality standards, both used desk and literature reviews, with WHO and UNAIDS focusing on facilitators and barriers to improving quality of health care for adolescents (26) but no search criteria was specified by Schools for Health in Europe (SHE) (27). In addition to the literature review, surveys and an analysis of national quality standards were used by WHO and United Nations Programme on HIV and AIDS (UNAIDS) (26). Once potential quality standards had been defined, both SHE (27) and WHO and UNAIDS (26) gathered consultation and peer review to prioritize which quality standards to include in the final set. Involving young people and caregivers was only mentioned by WHO and UNAIDS (26). No consistent approach to developing quality standards for child and adolescent mental health or health services was found in these documents.

*Objective (ii): To understand what methods have been recommended to develop quality standards for health services across different resource settings.*

Three guidance documents were found which recommended methods to develop quality standards for health services across different resource settings (25,28,29; see Table 2). All three guidance documents had different aims, from how to develop quality standards as a way to implement WHO's adolescent-friendly services (25), to a detailed process for how to develop clinical quality standards for high-priority health areas (28), to a high-level overview to develop quality standards for mental health (29). Not surprisingly, there was variation in the methods described. However, all included the importance of developing a working group in the preparatory work and then a process to develop the structure for the quality standards, including domains and how to measure them (25, 28, 29). Implementation plans were considered by two (25, 28), and the importance of involving patients and carers was highlighted by one (28). The level of detail outlined in the steps varied greatly, with materials to support the development of the quality standards in terms of PowerPoint presentation slides only provided by one (28). Hence, variations were found in the methods to develop quality standards proposed in these guidance materials.

### 3.2 Consultation with experts

Nine themes (of equal importance) emerged from the consultations with experts, to consider what methods should be used to develop quality standards for child and youth mental health services for use across the WHO European Region.

1. **Importance of using and developing robust methods**
  The importance of creating robust methods in order to develop quality standards that are themselves of high-quality and can be used across the WHO European Region was confirmed by the majority of the steering group. Robust methods were seen as the starting point to a high-quality end product which could be applied across multiple contexts.
2. **The importance of evidence and scientific rigor in all the processes and outputs**
  Using existing evidence and maintaining scientific rigor throughout was seen as essential to developing quality standards that are reflective of what high-quality care looks like to a broad range of stakeholders.
3. **Pragmatic approach**
  Whilst maintaining scientific rigor was essential, so was developing methods which would be feasible within the time and resource limits of the project. Hence, it was recognized that scientific rigor may need to be balanced with pragmatism. For example, it was mentioned that in order to develop quality standards that would be representative of the whole WHO European Region, a qualitative approach would be ideal across all countries. However due to resource limitations, a more rapid or mixed methods approach could be used (e.g., quantitative surveys).
4. **Clearly defining the scope and aims of the quality standards from the outset**
  The importance of defining the scope and aims of the quality standards from the outset was discussed. Questions included what level of the health system they should be aimed at, how it would fit into the wider health system such as primary health care, how they could fit outside of the health system (e.g., in criminal justice, education, social care), what kind of service would be aimed at, and who would be the main implementers of the quality standards.
5. **User engagement at the center of the development of the quality standards**
  The expert steering group members highlighted the importance to include young people at every step of the development of the quality standards, and ensure that caregiver and user voices are represented in the quality standards themselves. Challenges to engaging with users under the age of 18 years old were discussed, and ways to ensure emotional and physical safety of users when engaged in the process.
6. **Importance of wide stakeholder engagement in the development of the quality standards**.
  In addition to user engagement, the importance of including feedback from wider community members such as teachers, parents, social workers and the general public was highlighted, to develop a representative definition of high-quality care.
7. **The challenge of getting representation from across the Region**
  It was emphasized that efforts must be taken to ensure feedback from across the whole of the Region in the development of the quality standards, with a particular focus on non-English speaking countries, those that have not already established quality standards, and those which have fewer resources.
8. **To consider implementation from the outset and throughout the process**
  Potential challenges for implementation included getting buy-in from countries, engaging those who already have quality standards for child and adolescent mental health, communicating the quality standards to the public, and ensuring an efficient and sustained implementation process. Potential ways to overcome these challenges included developing a self-assessment tool, conducting a pilot implementation, engaging with civil society and service providers to advocate for implementation in a “bottom-up” approach, and engaging with Ministries of Health to implement in a “top-down” approach.
9. **Practical sharing of consultation methods to get feedback from citizens, users and stakeholders**
  Discussions and materials were shared to aid practical consultation from key stakeholders, including the use of case studies, treatment pathways, creative methods, focus groups, surveys, survey questions and photos.

### 3.3 Resulting methods for WHO quality standards for child and youth mental health services

Based on results from the desk searches (section 1 of results) and consultations with experts (section 2 of results), as well as the previously conducted rapid review, a seven-step methodology to develop quality standards for child and youth mental health services in the WHO European Region was developed (see Table 3). These methods aimed to align with the themes from consultations with experts as well as taking inspiration from the findings of the desk review and rapid review.

**Table 3:** Methods to develop WHO quality standards for child and youth mental health services.

Step	Information about the step
Step 1: Develop steering group	**Aim**: a group of people who can guide the rationale and purpose of the quality standards, the process to develop the quality standards, and provide feedback on initial versions of the quality standards.
	**Methods**: identify key stakeholders. This can be done through consultations, advertisements, and asking through networks. Key stakeholders include those: - With expertise in the process to develop quality standards, who can guide and provide feedback on the methods (e.g., those with experience of having previously developed quality standards). - With expertise of the context in which the quality standards will be implemented (e.g., Ministries of Health, child and adolescent mental health services). - With lived experience expertise of context in which the quality standard will be implemented - Who will support the implementation of the quality standards. **Output**: list of people to be on the steering group.
Step 2: Develop clear rationale, aim and purpose for quality standards	**Aim**: to have a clear and shared rationale, purpose and aim for the quality standards.
	**Methods**: interactive workshop with the steering group and any other key stakeholders of relevance.
	**Output**: A written document outlining: • Rationale for quality standards • Purpose • Context • Users • Usability/implementation • Outputs **Notes**: consider implementation of the quality standards from the outset, and usability of the end product.
Step 3: Rigorous methods to understand what high-quality care looks like for child and youth mental health services across the WHO European Region	i) *Review previously developed quality standards (country, multi-country, Regional and global)*
	**Aim:** to understand the definition of high-quality child and adolescent mental health care as defined through quality standards across the WHO European Region.
	**Methods**: scientific literature searches, online searches, and consultations.
	- Desk review of published research into quality standards for child and youth mental health services - Review of Ministry of Health websites, international organizations - Call-out to stakeholders
	**Output**: A list of quality statements/standards for child and youth mental health services taken from other quality standards.
	**Notes**: include only those that relate to the relevant service type and client group.
	ii) *Review of literature already published on feedback on child and adolescent mental health services from the perspective of users, carers/parents and providers in the health services.*
	**Aim:** to understand what research exists looking into user (children and young people; their caregivers) and provider feedback for high-quality child and youth mental health services across the Region.
	**Methods**: literature searches (systematic, rapid, non-systematic).
	**Output**: A list of domains which have been linked with high-quality care, and those which have been linked with low-quality care.
	**Notes**: look into whether any other systematic literature reviews already exist to save time.
	iii) *Stakeholder consultations on what high-quality care should look like for child and youth mental health services*
	**Aim**: to understand what high-quality care looks like for child and youth mental health services from key stakeholders (users and providers of child and youth mental health services).
	**Output**: A list of domains which have been linked with high-quality care for child and youth mental health services from groups of key stakeholders.
	**Methods**: - In-person small group discussions—with young people, citizens, service users, service providers, policy makers. - Survey—open to the public
	**Notes**: consider how to support young people with lived experience of mental health services in case discussions trigger distress; in person consultations and surveys can be used to supplement information from countries which are not represented in research findings.
Step 4: Analyze and consolidate findings from step 3	**Aim**: to bring together key themes on what high-quality care looks like for child and youth mental health services
	**Output**: a list of key themes of what high-quality care looks like based on results from step 3
	**Methods**: narrative analysis, thematic analysis.
Step 5—Prioritize quality standard themes/statements for inclusion in initial set of quality standards	**Aim:** to gather feedback from a wide range of stakeholders on what quality standards should be included.
	**Output**: a list of prioritized quality statements/themes.
	**Methods**: In-person prioritization exercises across multiple stakeholder groups—including young people, mental health service providers, academics, Ministry of Health/leaders in countries, caregivers.
Step 6—feedback and update quality standards in iterative cycles	**Aim:** to gather feedback on the initial set of quality standards in iterative cycles from a wider audience.
	**Output**: new set of quality standards updated based on iterative cycles of feedback from sequentially bigger groups of stakeholders.
	**Methods**: - Gathering verbal feedback through small in-person consultations with key stakeholders. - Gathering verbal feedback through larger in-person consultations with key stakeholders. - Gathering written and verbal feedback through disseminating initial version of quality standards to stakeholders. - Gathering written feedback through disseminating initial version of quality standards to the public.
	**Notes**: consider how to engage members of the public and young people.
Step 7—pilot implementation	**Aim:** to understand key barriers and facilitation to implementation and thus inform development of implementation support tools/resources.
	**Output**: written document outlining proposed steps for implementation based on results from the pilot.
	**Methods**: - Working with a small number of interested countries to understand key barriers and facilitators to implementation. - Tailored support to facilitate the implementation. **Notes**: consider engaging with implementation experts for this phase.

As can be seen from Table 3, the first steps are to define the steering group and the implementation aims of the quality standards (steps 1 and 2). Next, the definition of high-quality care and hence initial themes of what should be included in the quality standards needs to be developed, including perspectives from a wide range of stakeholders and the evidence (steps 3–4). Consultation across a wide range of stakeholders is then used to iteratively update the themes for high-quality care (steps 5 and 6) until an initial set of quality standards is developed. The final phase is to pilot their implementation (step 7).

## 4 Discussion

This research showed that there is not a consistent approach used or recommended to developing quality standards for child and youth mental health/health settings in the grey literature. A unified procedure to develop quality standards for child and youth mental health services is needed to ensure that quality standards do not differ in what and whose definition of high-quality care they represent. Furthermore, having a unified approach to quality improvement provides opportunity for more efficient use of resources (30), something particularly pertinent in child and youth mental health services which are often under-resourced. This finding is consistent with wider research which has called for unified approach to develop health quality indicators (21).

Our desk reviews found that published practical resources to support the development of quality standards for health services were limited, making it challenging for those who do wish to follow guidance or replicate methods to develop quality standards. This points to a need for more practical resources and sharing of information to support quality standard development, for example on survey questions, how to balance pragmatism and rigorous scientific rigor, and how to meaningfully involve people with lived experience.

There were limitations in our methods. Our desk searches were not systematic literature reviews, instead we took a programmatic approach due to time and resource constraints. We focused specifically on the development of quality standards relating to child and youth mental health/health services from international organizations. This means that our searches may have been incomplete. The methods taken to gather feedback from key stakeholders were not scientifically rigorous. We did not record and transcribe interviews, instead relying on notes from meetings. To mitigate this, the resulting methods document was reviewed by all stakeholders to provide them with the opportunity to input anything else which may have been missing.

However, the process did allow for us to meet the aims of the research, that is to develop a methodology grounded in available evidence for developing quality standards for child and adolescent mental health across the WHO European Region.

Quality standards define how care should ideally be provided, and quality assessment and improvement efforts centre around implementing these quality standards. Hence it is essential that the quality standards developed accurately define high-quality care. If the original definition is not evidence-based or agreed upon by multiple stakeholders, then subsequent quality assessment and improvement efforts may be misguided. Standardization can also reduce waste, add value and save resources. Future directions include the need to develop a consistent methodology to create quality standards which are evidence-informed and represent views of multiple stakeholders, and to understand how quality improvement efforts can reduce waste in low-resource settings.

Engaging with citizens and users is an established strategy to improve the quality of care (31). Hence the engagement of citizens and users should be a requirement when developing quality of care standards, to ensure that services and quality improvement efforts are embedded in the needs and preferences of the users. Further research can support this through understanding what the needs and preferences are of citizens and users when accessing child and youth mental health care. In particular, more research is needed from low-resource and middle-income countries.

Piloting the quality standards was emphasized as a key step to understand and adapt implementation based on barriers and facilitators. It is likely that the implementation of the quality standards will need to be adapted based on the context of implementation, including the resources available, whether a continuous culture of quality improvement exists, as well as individual staff motivation. Given the lack of published research on how to improve quality of child and adolescent mental health services (32), it is recommended that implementation efforts include a strong research component, and aim to understand what approaches are the most effective as well as any unintended negative consequences, across all resource settings.

In order to effectively improve the quality of child and youth mental health care, it is essential to first develop a shared definition of high-quality care through quality standards. The extent to which this definition will be representative of multiple stakeholders and the evidence depends on the methods used. By detailing the methods used to develop quality standards for child and youth mental health services, it is hoped that this paper will provide direction to others wishing to develop quality standards and spark further research in this area. Proposed future directions include developing a unified approach to developing quality standards, more research to understand the preferences and needs of users of child and youth mental health services, how to best improve quality of care and patient safety in child and youth mental health care, practical resources on how to develop quality standards, and to use quality improvement to guide more efficient use of resources, particularly in under-resourced settings.

## Data Availability

The original contributions presented in the study are included in the article/Supplementary Material, further inquiries can be directed to the corresponding author.
